# The association between higher FFAs and high residual platelet reactivity among CAD patients receiving clopidogrel therapy

**DOI:** 10.3389/fcvm.2023.1115142

**Published:** 2023-05-26

**Authors:** Zehao Zhao, Shutong Dong, Tienan Sun, Kangning Han, Xin Huang, Meishi Ma, Shiwei Yang, Yujie Zhou

**Affiliations:** ^1^ Department of Cardiology, Beijing Anzhen Hospital, Capital Medical University, Beijing, China; ^2^ Beijing Institute of Heart, Lung and Blood Vessel Disease, Beijing, China; ^3^ Beijing Key Laboratory of Precision Medicine of Coronary Atherosclerotic Disease, Clinical Center for Coronary Heart Disease, Capital Medical University, Beijing, China

**Keywords:** free fatty acids (FFA), coronary artery disease, clopidogrel, platelet reactivity, thromboelastogram, percutaneous coronary intervention

## Abstract

**Background:**

Metabolic abnormalities are associated with the occurrence, severity, and poor prognosis of coronary artery disease (CAD), some of which affect the antiplatelet efficacy of clopidogrel. Free fatty acids (FFAs) is a biomarker for metabolic abnormalities, and elevated FFAs is observed among CAD patients. Whether FFAs enhances residual platelet reactivity induced by adenosine diphosphate (ADP) while using clopidogrel was unknown. The purpose of our study is exploring the issue.

**Method:**

Current study included 1,277 CAD patients using clopidogrel and used logistic regression to detect whether the higher level of FFAs is associated with high residual platelet reactivity (HRPR). We additionally performed subgroup and sensitivity analyses to evaluate the stability of the results. We defined HRPR as ADP-induced platelet inhibition rate (ADP_i_) < 50% plus ADP-induced maximum amplitude (MA_ADP_) > 47 mm.

**Results:**

486 patients (38.1%) showed HRPR. The proportion of HRPR among patients with higher FFAs (>0.445 mmol/L) is greater than among patients with lower FFAs (46.4% vs. 32.6%, *P* < 0.001). Multivariate logistic regression demonstrated that higher FFAs (>0.445 mmol/L) is independently associated with HRPR (adjusted OR = 1.745, 95% CI, 1.352–2.254). After subgroup and sensitivity analyses, the results remained robust.

**Conclusion:**

The higher level of FFAs enhances residual platelet reactivity induced by ADP and is independently associated with clopidogrel HRPR.

## 1. Introduction

CAD is a common cardiovascular disease, which is commonly caused by atherosclerotic plaques. Coronary artery plaque rupture triggers platelet adhesion to exposed sub-endothelial matrix proteins, platelet activation, and platelet aggregation, which might cause severe ischemic events (1). Antiplatelet therapy is a powerful strategy for preventing thrombosis and ischemic events. Dual antiplatelet therapy (DAPT), composed of oral P2Y12 receptor antagonist and aspirin, is an important component of secondary prevention for CAD patients. By virtue of lower bleeding risk (2, 3) and fewer adverse effects, clopidogrel is still indispensable in the era of emerging novel P2Y12 receptor antagonists. Clopidogrel is recommended for application in patients suffering from stable coronary artery disease (SCAD) (4, 5). Many factors affect the pharmacodynamics of clopidogrel, such as genetic factors, drug-drug interaction, and biological and clinical factors (6). A notable proportion of CAD patients using clopidogrel therapy present HRPR and are more likely to experience adverse events after percutaneous coronary intervention (PCI) (7).

Elevated FFAs is often observed among patients with metabolic abnormalities, such as diabetes mellitus, obesity, hypertension, and so on (8, 9). Metabolic abnormalities are proven risk factors for CAD (10), some of which were found to be associated with HRPR (11, 12). FFAs is also a biomarker for coronary artery thrombosis (13, 14), and is associated with the occurrence, severity, and poor prognosis of CAD (15–17). Whether FFAs affect clopidogrel's pharmacodynamics remained unclear. We conducted a cross-sectional study by collecting clinical information from CAD patients scheduled for elective PCI. The aim is to detect whether FFAs affect ADP-induced platelet reactivity in CAD patients receiving clopidogrel and evaluate the predictive value of FFAs for HRPR.

## 2. Method

### 2.1. Study design and population

Based on a prospectively collected database, we conducted a cross-sectional study to assess whether FFAs affects residual platelet reactivity while receiving clopidogrel therapy. This research consecutively enrolled 1,380 SCAD patients who underwent elective PCI from January 2019 to December 2019. All patients had available FFAs measurements and had received guideline-recommended preoperative clopidogrel therapy [maintenance dose (75 mg, once daily) for at least five days prior to PCI or loading dose (300 mg) of clopidogrel at least 12 h prior to PCI]. The exclusion criteria included: (1) incomplete baseline data, (2) receiving other P2Y12 inhibitors besides clopidogrel, (3) intolerance of DAPT consisting of aspirin and clopidogrel (such as drug allergy and BARC ≥ 3 bleeding events), (4) platelet count (PLT) > 400 or < 50 × 10^9^/L, (5) severe renal or hepatic impairment [estimated glomerular filtration rate (eGFR) < 30 ml/min/1.73 m^2^ and/or alanine aminotransferase (ALT) > 2.5 times the normal upper limit], (6) other life-threatening diseases such as cancer.

### 2.2. Demographic, clinical, and laboratory information

Patients' information were documented by trained data collectors from electronic medical record system without knowledge of the current study's protocol. Demographic information included age and gender. Clinical data collected included heart rate, systolic and diastolic blood pressure, smoking status, body mass index (BMI), and medical history of hypertension, diabetes, dyslipidemia, and previous coronary revascularization (percutaneous or surgical). Current smoker was defined as self-reported regular tobacco use in the last 3 months. Hypertension was diagnosed based on a previous hypertension diagnosis, using an anti-hypertensive medication, or resting blood pressure ≥140/90 mmHg. Dyslipidemia was defined as having a previous diagnosis, currently undergoing lipid-lowering therapy, or having low-density lipoprotein cholesterol (LDL-C) > 4.1 mmol/L and/or high-density lipoprotein cholesterol (HDL-C) < 1.0 mmol/L and/or total cholesterol (TC) > 6.2 mmol/L and/or triglyceride (TG) > 2.3 mmol/L. Diabetes was defined as a self-reported previous diagnosis, receiving glucose-lowering therapy, or a new diagnosis based on current guidelines (18). Besides these, we collected laboratory measurements, including FFAs, routine blood indicators [white blood cell count (WBC), PLT, and hemoglobin (Hb)], liver function indicators [albumin (ALB), ALT, and aspartate aminotransferase (AST)], renal function indicators [creatinine and eGFR], fasting blood glucose (FBG), lipid indicators (TG, TC, HDL-C, and LDL-C), fibrinogen (FIB), uric acid (UA), high-sensitivity C-reactive protein (hs-CRP). eGFR was calculated using an online tool provided by Chronic Kidney Disease Epidemiology Collaboration (http://ckdepi.org/equations/gfr-calculator/). FFAs was measured using an automated biochemical analyzer with the enzymatic colorimetric method (Roche, COBAS 8000). To avoid the condition that FFAs is unable to efficiently reflect the abnormal metabolism due to interfering factors (such as physical activity, hunger state, and so on), blood samples were taken from the patient at a quiet state after 12 h of fasting. In addition, 768 patients among them had ever tested CYPC19*2, CYPC19*3, and CYPC19*17 alleles, and we collected these information by browsing the electronic medical record system.

### 2.3. Platelet function testing

On the first morning after PCI, nurses collected venous blood samples into vacutainer tubes containing lithium heparin and 3.2% trisodium citrate, and the thrombelastography (TEG) assay was carried out within 2 h using LEPU TEG System (CFMS, Beijing, China) to evaluate the efficacy of antiplatelet therapy. The ADP-induced thrombus formation process monitored by the TEG was reported as a series of coagulation parameters. Previous literature had described the detection principle in detail (19). The modified TEG system evaluated the effects of antiplatelet therapy action via the ADP and arachidonic acid pathways using four channels. The CFMS TEG system and automated analytical software measured the physical properties of clots. MA_ADP_ reflects the maximum intensity of ADP-induced clots consisting of fibrin and platelet in a heparinized whole blood sample. The maximum amplitude of thrombin-induced clot strength (MA_thrombin_) represented the aggregation capacity of platelet and fibrin induced by thrombin and the maximum amplitude of fibrin clot strength (MA_fibrin_) represented the aggregation capacity of only fibrin induced by reptilase and factor XIIIa. ADP_i_ was used to assess clopidogrel's efficacy in blocking the ADP pathway to inhibit platelet aggregation, which was calculated as $\mathrm{AD}P_{i}=\frac{MA_{\mathrm{ADP}}-MA_{\mathrm{fibrin}}}{MA_{\mathrm{thrombin}}-MA_{\mathrm{Fibrin}}}$. Based on current consensus (20) and previous studies conducted in China (21), we defined ADP-induced HRPR as ADP*_i_* < 50% plus MA_ADP_ > 47 mm in the current study.

### 2.4. Statistical methods

Continuous variables were expressed as the mean and standard deviation (SD) or the median and interquartile range (IQR) according to normal or non-normal distribution. Normality testing was performed using graphical methods and the Shapiro-Wilk test. Categorical variables were expressed as numbers and percentages (%). We presented baseline characteristics of patients with HRPR and non-HRPR. We used the Student's *t*-test, the Mann–Whitney test, or the chi-square test to compare differences between the two groups as appropriate. To compare residual platelet activity and other covariates among patients with high and low levels of FFAs, we additionally divided patients into two groups based on an FFAs level of 0.445 mmol/L. A receiver operating characteristic (ROC) curve was drawn to determine the optimum cut-off value (0.445 mmol/L) based on the Youden index. The exposure risk of FFA > 0.445 mmol/L was tested using a univariate logistic regression model and expressed as the odds ratio (OR) and 95% confidence interval (95% CI). To correct potential confounding factors, we constructed a series of multivariable logistic regression models in several stages. The choice of covariates was based on current statistical significance, previous literature, and clinical judgment. Model 1: adjusted for sex and age, Model 2: adjusted for the medical history of hypertension, diabetes, and previous PCI, current smoker, usage pattern of clopidogrel, and variables included in Model 1, and Model 3: adjusted for Hb, WBC, PLT, HDL-C, LDL-C, ALT, creatine, FIB, hs-CRP, and variables included in Model 2. Then, we performed subgroup analysis to test the stability of results and detect potential interaction. Interactions were tested by using an interaction test in logistic regression models. Furthermore, we additionally conducted sensitivity analyses using propensity score matching (PSM). Groups (HRPR and non-HRPR) were matched using 1:1 nearest neighbor-matching, within a caliper width of 0.2 of the standard deviation of the propensity score logit. The variables selected for the propensity score model included covariates in Model 3. In addition, we again conducted univariate and multivariable analyses among patients with the CYPC19 genotype and tested the stability of the results after adjusting for variables including clopidogrel's metabolizer status.

Statistical significance was determined with a 2-tailed *P*-value <0.05. SPSS Statistics 26.0 (SPSS, Inc., Chicago, IL, USA) was used for the statistical analysis.

## 3. Results

### 3.1. Study population

**Figure 1:**
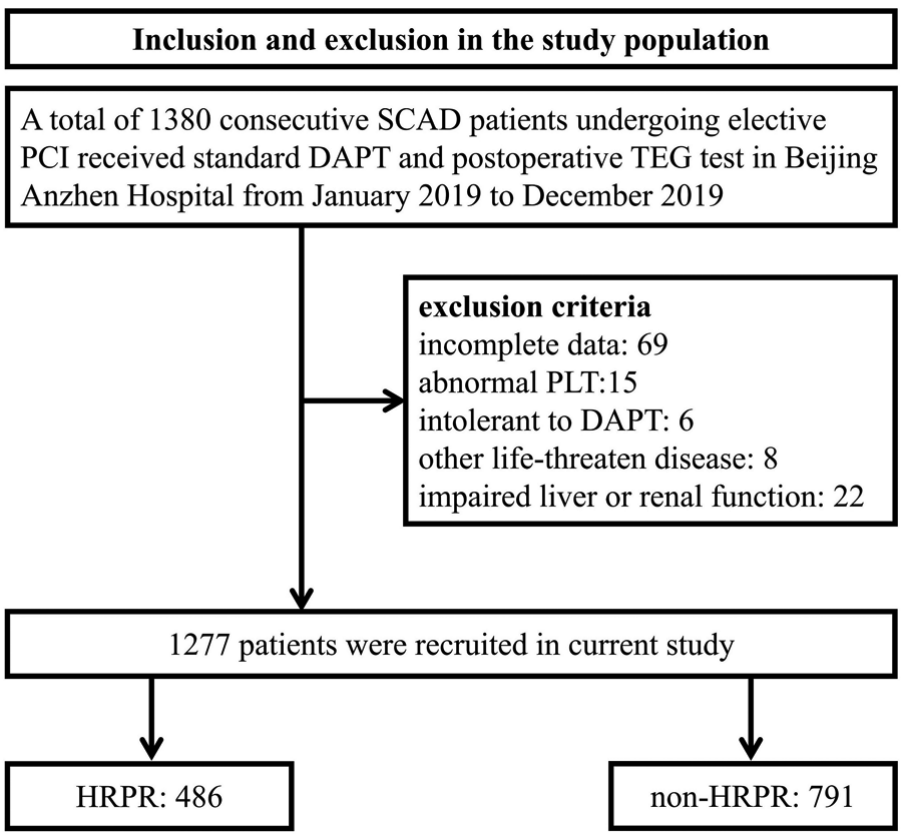
Inclusion and exclusion in the study population. SCAD, stable coronary artery disease; PCI, percutaneous coronary intervention; DAPT, Dual antiplatelet therapy; TEG, thromboelastography; PLT, platelet count; HRPR, high residual platelet reactivity.

As shown by Figure 1, after careful screening, a total of 1,277 patients satisfied the inclusion and exclusion criteria and were enrolled in the current study, of which 486 patients (38.1%) showed HRPR. The mean age was 61.2 ± 9.5 years and 337 patients (26.4%) were female. Baseline information were complete. In Table 1, baseline characteristics were shown using stratification based on HRPR and non-HRPR. According to univariate comparison, patients with HRPR were older, and more frequently female. Patients with HRPR had a significantly higher proportion of diabetes and a significantly lower proportion of previous PCI. The proportion of using 300 mg loading clopidogrel or using daily 75 mg clopidogrel more than 5 days before PCI is balanced between groups. In terms of laboratory information, patients with HRPR had higher levels of FFAs (0.46 ± 0.22 vs. 0.41 ± 0.21, *P* < 0.001), PLT, HDL-C, LDL-C, FIB, FBG, and hs-CRP, and had lower levels of Hb, WBC, creatine, and eGFR. Additionally, we found CYPC19 genotype distributions between the two groups were different among 768 patients who had tested CYPC19*2, CYPC19*3, and CYPC19*17 alleles (Supplementary material S1, Table S1). The proportions of intermediate metabolizers and poor metabolizers are higher among patients with HRPR.

**Table 1:** Baseline characteristics in patients with and without HRPR.

Variables	Total (*n* = 1,277)	non-HRPR (*n* = 791)	HRPR (*n* = 486)	*P*
Age (years), Mean ± SD	61.2 ± 9.5	60.3 ± 9.6	62.8 ± 9.1	<0.001
Female, *n* (%)	337 (26.4%)	151 (19.1%)	186 (38.3%)	<0.001
BMI (kg/m^2^), Mean ± SD	25.8 ± 3.2	25.8 ± 3.2	25.7 ± 3.3	0.41
SBP (mmHg), Mean ± SD	129 ± 16	128.3 ± 16.1	130 ± 15.8	0.074
DBP (mmHg), Mean ± SD	75.3 ± 11.1	75.4 ± 11.1	75 ± 11.2	0.511
Heart rate (bpm), Mean ± SD	69.6 ± 7.7	69.6 ± 7.7	69.6 ± 7.8	0.984
Current smoker, *n* (%)	331 (25.9%)	219 (27.7%)	112 (23.0%)	0.076
Hypertension, *n* (%)	794 (62.2%)	478 (60.4%)	316 (65.0%)	0.113
Diabetes, *n* (%)	445 (34.8%)	252 (31.9%)	193 (39.7%)	0.005
Dyslipidemia, *n* (%)	694 (54.3%)	440 (55.6%)	254 (52.3%)	0.266
Previous CABG, *n* (%)	30 (2.3%)	16 (2.0%)	14 (2.9%)	0.428
Previous PCI, *n* (%)	372 (29.1%)	249 (31.5%)	123 (25.3%)	0.022
Clopidogrel loading, *n* (%)	660 (51.7%)	398 (50.3%)	262 (53.9%)	0.212
FFAs (mmol/L), Mean ± SD	0.4 ± 0.2	0.4 ± 0.2	0.5 ± 0.2	<0.001
WBC (×10^9^/L), Mean ± SD	6.7 ± 1.9	6.9 ± 1.9	6.5 ± 1.8	<0.001
PLT (×10^9^/L), Mean ± SD	212.7 ± 54	205.9 ± 53.8	223.9 ± 52.5	<0.001
Hb (g/L), Mean ± SD	140.9 ± 15.9	143.2 ± 15.7	137.1 ± 15.4	<0.001
ALT (mmol/L), Median [IQR]	21.0 [15.0, 29.0]	21.0 [15.0, 29.0]	20.0 [15.0, 30.0]	0.314
AST (mmol/L), Median [IQR]	21.0 [18.0, 25.0]	21.0 [18.0, 25.0]	21.0 [18.0, 26.0]	0.444
ALB (mmol/L), Mean ± SD	42.4 ± 3.6	42.4 ± 3.4	42.4 ± 3.8	0.892
eGFR (ml/min/1.73 m^2^), Median [IQR]	94.7 [86.2, 101.2]	95.3 [87.0, 102.1]	93.4 [84.9, 99.9]	0.001
Creatine (µmol/L), Mean ± SD	71.7 ± 17.9	72.5 ± 17.3	70.5 ± 18.9	0.049
UA (µmol/L), Mean ± SD	350.7 ± 86.8	352.9 ± 83.9	347.2 ± 91.4	0.27
TG (mmol/L), Mean ± SD	1.6 ± 1.2	1.6 ± 1.2	1.6 ± 1.0	0.465
TC (mmol/L), Mean ± SD	4 ± 1	3.9 ± 0.9	4.1 ± 1.0	<0.001
HDL-C (mmol/L), Mean ± SD	1.1 ± 0.3	1.1 ± 0.2	1.1 ± 0.3	0.012
LDL-C (mmol/L), Mean ± SD	2.3 ± 0.8	2.3 ± 0.8	2.4 ± 0.9	0.006
FBG (mmol/L), Median [IQR]	5.7 [5.1, 6.9]	5.6 [5.0, 6.9]	5.7 [5.1, 6.9]	0.077
FIB (g/L), Mean ± SD	3.2 ± 0.6	3.1 ± 0.6	3.3 ± 0.6	<0.001
hs-CRP (mmol/L), Median [IQR]	1.2 [0.5, 3.1]	1.1 [0.4, 2.7]	1.4 [0.7, 3.7]	<0.001
MA_ADP_ (mm), Median [IQR]	43.4 [34.9, 51.3]	37.1 [29.6, 41.8]	53.8 [50, 58.4]	<0.001
ADP_i_ (%), Mean ± SD	37.1 ± 23.6	50.3 ± 19.4	15.7 ± 10.5	<0.001

HRPR, high residual platelet reactivity; BMI, body mass index; SBP, systolic blood pressure; DBP, diastolic blood pressure; PCI, percutaneous coronary intervention; CABG, coronary artery bypass graft; FFAs, free fatty acids; WBC, white blood cell count; PLT, platelet count; Hb, hemoglobin; ALT, alanine aminotransferase; AST, aspartate aminotransferase; ALB, albumin; eGFR, estimated glomerular filtration rate; UA, uric acid; TC, total cholesterol; TG, triglyceride; LDL-C, low density lipoprotein cholesterol; HDL-C, high density lipoprotein cholesterol; FBG, fasting blood glucose; FIB, fibrinogen; hs-CRP, high-sensitivity C-reactive protein; MA_ADP_, Maximum amplitude of ADP-induced clot strength; ADP_i_, ADP-induced platelet inhibition rate.

### 3.2. Platelet aggregation and other covariates among different levels of FFAs

**Figure 2:**
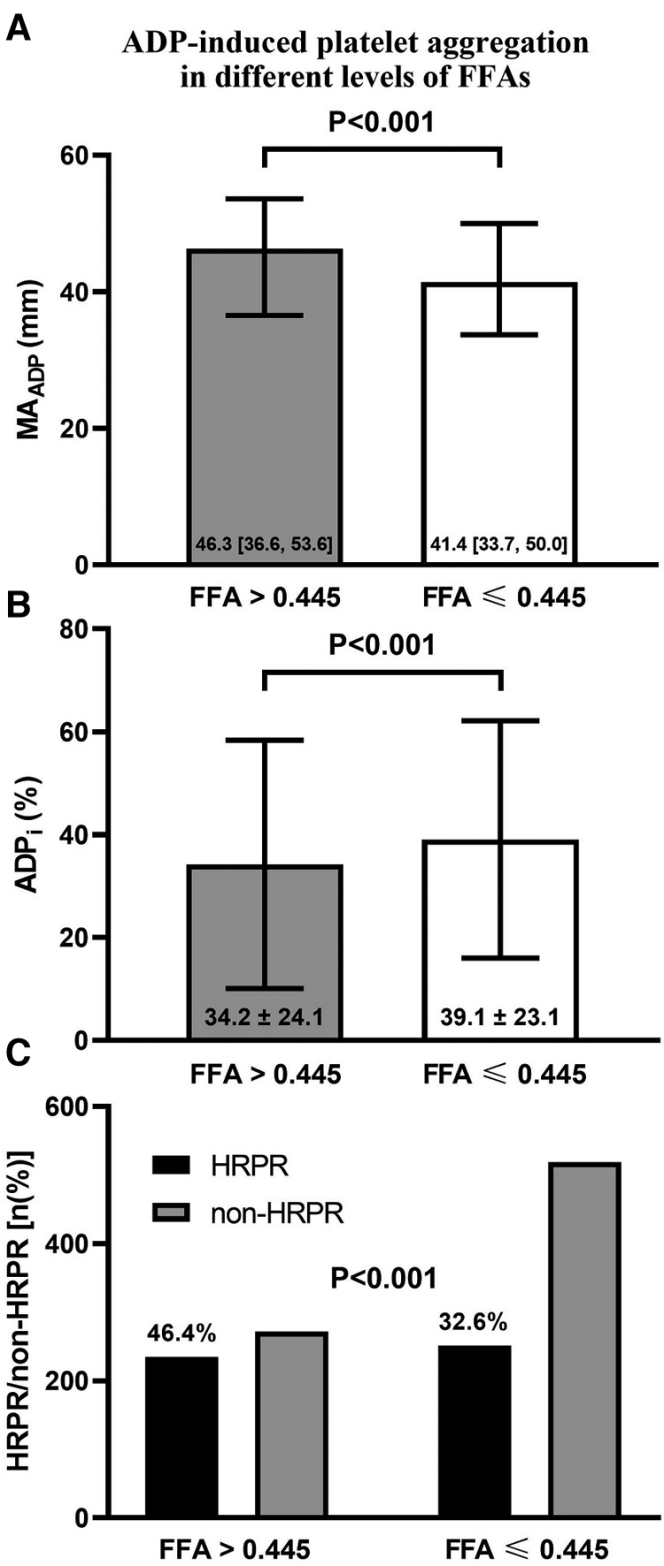
ADP-induced platelet aggregation in different levels of FFAs. (**A**) ADP-induced platelet inhibition rate (ADP_i_), (**B**) the maximum amplitude of ADP-induced clot strength (MA_ADP_) and (**C**) the frequency of HRPR in patients with different FFAs level.

We divided all patients included in the current study into two groups based on an FFAs level of 0.445 mmol/L. The cut-off point was determined by detecting the point with the optimum Youden index from the ROC curve (Supplementary Material S3). As shown in Table 2, patients with higher FFAs were more often female and had a significantly higher proportion of diabetes and hypertension. Besides these, patients with higher FFAs had higher levels of Hb, aminotransferase, lipid indicators, FBG, FIB, and hs-CRP, and lower creatine. Notably, there were significant differences in TEG parameters between groups with higher and lower FFAs. As shown in Figure 2, there existed higher MA_ADP_ and lower ADP_i_ among patients with higher FFAs (the median value of MA_ADP_: 46.3 [36.6, 53.6] vs. 41.4 [33.7, 49.95], *P* < 0.001; the mean value of ADP_i_: 34.21 ± 24.12 vs. 39.06 ± 23.06, *P* < 0.001), and the proportion of HRPR among patients with higher FFAs was larger (46.4% vs. 32.6%, *P* < 0.001).

**Table 2:** Platelet aggregation and other covariates among different levels of FFAs.

Variables	Total (*n* = 1,277)	FFAs ≤ 0.445 (*n* = 770)	FFAs > 0.445 (*n* = 507)	*P*
Age (years), Mean ± SD	61.2 ± 9.5	60.9 ± 9.4	61.7 ± 9.6	0.151
Female, *n* (%)	337 (26.4%)	184 (23.9%)	153 (30.2%)	0.015
BMI (kg/m^2^), Mean ± SD	25.8 ± 3.2	25.5 ± 3	26.1 ± 3.4	<0.001
SBP (mmHg), Mean ± SD	129 ± 16	128.1 ± 15.9	130.2 ± 16.1	0.022
DBP (mmHg), Mean ± SD	75.3 ± 11.1	74.5 ± 10.9	76.4 ± 11.5	0.004
Heart rate (bpm), Mean ± SD	69.6 ± 7.7	69.6 ± 7.8	69.6 ± 7.7	0.97
Current smoker, *n* (%)	331 (25.9%)	214 (27.8%)	117 (23.1%)	0.069
Hypertension, *n* (%)	794 (62.2%)	448 (58.2%)	346 (68.2%)	<0.001
Diabetes, *n* (%)	445 (34.8%)	235 (30.5%)	210 (41.4%)	<0.001
Dyslipidemia, *n* (%)	694 (54.3%)	438 (56.9%)	256 (50.5%)	0.029
previous CABG, *n* (%)	30 (2.3%)	23 (3.0%)	7 (1.4%)	0.096
previous PCI, *n* (%)	372 (29.1%)	224 (29.1%)	148 (29.2%)	1
Clopidogrel loading, n(%)	660 (51.7%)	404 (52.2%)	258 (50.9%)	0.644
FFAs (mmol/L), Mean ± SD	0.4 ± 0.2	0.3 ± 0.1	0.6 ± 0.2	<0.001
WBC (×10^9^/L), Mean ± SD	6.7 ± 1.9	6.8 ± 2	6.7 ± 1.7	0.358
PLT (×10^9^/L), Mean ± SD	212.7 ± 54	212.6 ± 54.9	212.9 ± 52.5	0.933
Hb (g/L), Mean ± SD	140.9 ± 15.9	139.9 ± 15.9	142.3 ± 15.7	0.01
ALT (mmol/L), Median [IQR]	21.0 [15.0, 29.0]	20.0 [15.0, 28.0]	22.0 [16.0, 30.5]	0.025
AST (mmol/L), Median [IQR]	21.0 [18.0, 25.0]	20.0 [17.0, 24.0]	22.0 [18.0, 26.4]	<0.001
ALB (mmol/L), Mean ± SD	42.4 ± 3.6	41.6 ± 3.4	43.5 ± 3.5	<0.001
eGFR (ml/min/1.73 m^2^), Median [IQR]	94.7 [86.2, 101.2]	95.0 [86.4, 101.6]	94.1 [86.1, 100.8]	0.312
Creatine (µmol/L), Mean ± SD	71.7 ± 17.9	72.5 ± 19.1	70.5 ± 16	0.043
UA (µmol/L), Mean ± SD	350.7 ± 86.8	349.8 ± 83.8	352.1 ± 91.3	0.659
TG (mmol/L), Mean ± SD	1.6 ± 1.2	1.6 ± 1.1	1.7 ± 1.2	0.026
TC (mmol/L), Mean ± SD	4.0 ± 1.0	3.9 ± 0.9	4.2 ± 1.0	<0.001
HDL-C (mmol/L), Mean ± SD	1.1 ± 0.3	1.1 ± 0.2	1.1 ± 0.3	<0.001
LDL-C (mmol/L), Mean ± SD	2.3 ± 0.8	2.3 ± 0.8	2.4 ± 0.9	0.005
FBG (mmol/L), Median [IQR]	5.7 [5.1, 6.9]	5.6 [5.0, 6.6]	5.8 [5.1, 7.3]	<0.001
FIB (g/L), Mean ± SD	3.2 ± 0.6	3.1 ± 0.6	3.2 ± 0.6	0.039
hs-CRP (mmol/L), Median [IQR]	1.2 [0.5, 3.1]	1.1 [0.5, 2.9]	1.3 [0.6, 3.3]	0.012
ADP_MA_ (mm), Median [IQR]	43.4 [34.9, 51.3]	41.4 [33.7, 50.0]	46.3 [36.6, 53.6]	<0.001
ADP_i_ (%), Mean ± SD	37.1 ± 23.6	39.1 ± 23.1	34.2 ± 24.1	<0.001
HRPR, *n* (%)	486 (38.1%)	251 (32.6%)	235 (46.4%)	<0.001

HRPR, high residual platelet reactivity; BMI, body mass index; SBP, systolic blood pressure; DBP, diastolic blood pressure; PCI, percutaneous coronary intervention; CABG, coronary artery bypass graft; FFAs, free fatty acids; WBC, white blood cell count; PLT, platelet count; Hb, hemoglobin; ALT, alanine aminotransferase; AST, aspartate aminotransferase; ALB, albumin; eGFR, estimated glomerular filtration rate; UA, uric acid; TC, total cholesterol; TG, triglyceride; LDL-C, low density lipoprotein cholesterol; HDL-C, high density lipoprotein cholesterol; FBG, fasting blood glucose; FIB, fibrinogen; hs-CRP, high-sensitivity C-reactive protein; MA_ADP_, Maximum amplitude of ADP-induced clot strength; ADP_i_, ADP-induced platelet inhibition rate.

### 3.3. Effect of FFAs on residual platelet reactivity

FFAs was recategorized as a dichotomous variable (FFAs > 0.445 and FFAs ≤ 0.445). To evaluate the effect of FFAs on ADP-induced residual platelet reactivity while using clopidogrel, we constructed univariable and multivariable logistic regression models including FFAs. As shown in Table 3, crude OR is 1.786 (1.419–2.250), which might suggest an association between higher FFAs and HRPR. As mentioned in the previous section, we constructed a series of multivariable logistic regression models to adjust for potential confounders and demonstrated higher FFAs as an independent factor associated with HRPR (model3: OR 1.745, 95%CI, 1.352–2.254). Besides this, we also found that age, female, higher PLT, lower Hb, lower WBC, higher ALT, and higher FBG were independently associated with HRPR.

**Table 3:** Univariable and multivariable logistic regression models.

Variables	OR (95% CI)			
	Crude Model	Model 1	Model 2	Model 3
FFAs > 0.445 mmol/L	1.786 (1.419–2.250)	1.710 (1.349–2.166)	1.687 (1.327–2.144)	1.745 (1.352–2.254)
Age	–	1.020 (1.007–1.033)	1.023 (1.009–1.036)	1.027 (1.012–1.042)
Female	–	2.381 (1.834–3.092)	2.432 (1.846–3.205)	1.613 (1.117–2.328)
Diabetes	–	–	1.261 (0.984–1.616)	1.267 (0.975–1.647)
Hypertension	–	–	0.997 (0.778–1.278)	1.000 (0.768–1.302)
Current smoker	–	–	1.163 (0.868–1.557)	1.213 (0.892–1.649)
Previous PCI	–	–	0.719 (0.549–0.942)	0.798 (0.600–1.061)
Clopidogrel loading	–	–	1.076 (0.846–1.369)	1.151 (0.895–1.480)
Hb	–	–	–	0.985 (0.976–0.995)
WBC	–	–	–	0.812 (0.751–0.879)
PLT	–	–	–	1.006 (1.003–1.008)
LDL-C	–	–	–	1.112 (0.949–1.305)
HDL-C	–	–	–	1.095 (0.651–1.842)
ALT	–	–	–	1.015 (1.006–1.025)
Creatine	–	–	–	1.002 (0.994–1.009)
FIB	–	–	–	1.849 (1.419–2.409)
hs-CRP	–	–	–	0.986 (0.955–1.019)

Model 1: adjusted for sex and age, Model 2: adjusted for the medical history of hypertension, diabetes, and previous PCI, current smoker, usage pattern of clopidogrel, and variables included in Model 1, and Model 3: adjusted for Hb, WBC, PLT, HDL-C, LDL-C, ALT, creatine, FIB, hs-CRP, and variables included in Model 2. OR, Odds ratio; CI, Confidence interval; HRPR, high residual platelet reactivity; FFAs, free fatty acids; PCI, percutaneous coronary intervention; Hb, hemoglobin; WBC, white blood cell count; PLT, platelet count; LDL-C, low density lipoprotein cholesterol; HDL-C, high density lipoprotein cholesterol; ALT, alanine aminotransferase; FIB, fibrinogen; hs-CRP, high-sensitivity C-reactive protein.

### 3.4. Subgroup and sensitivity analyses

**Figure 3:**
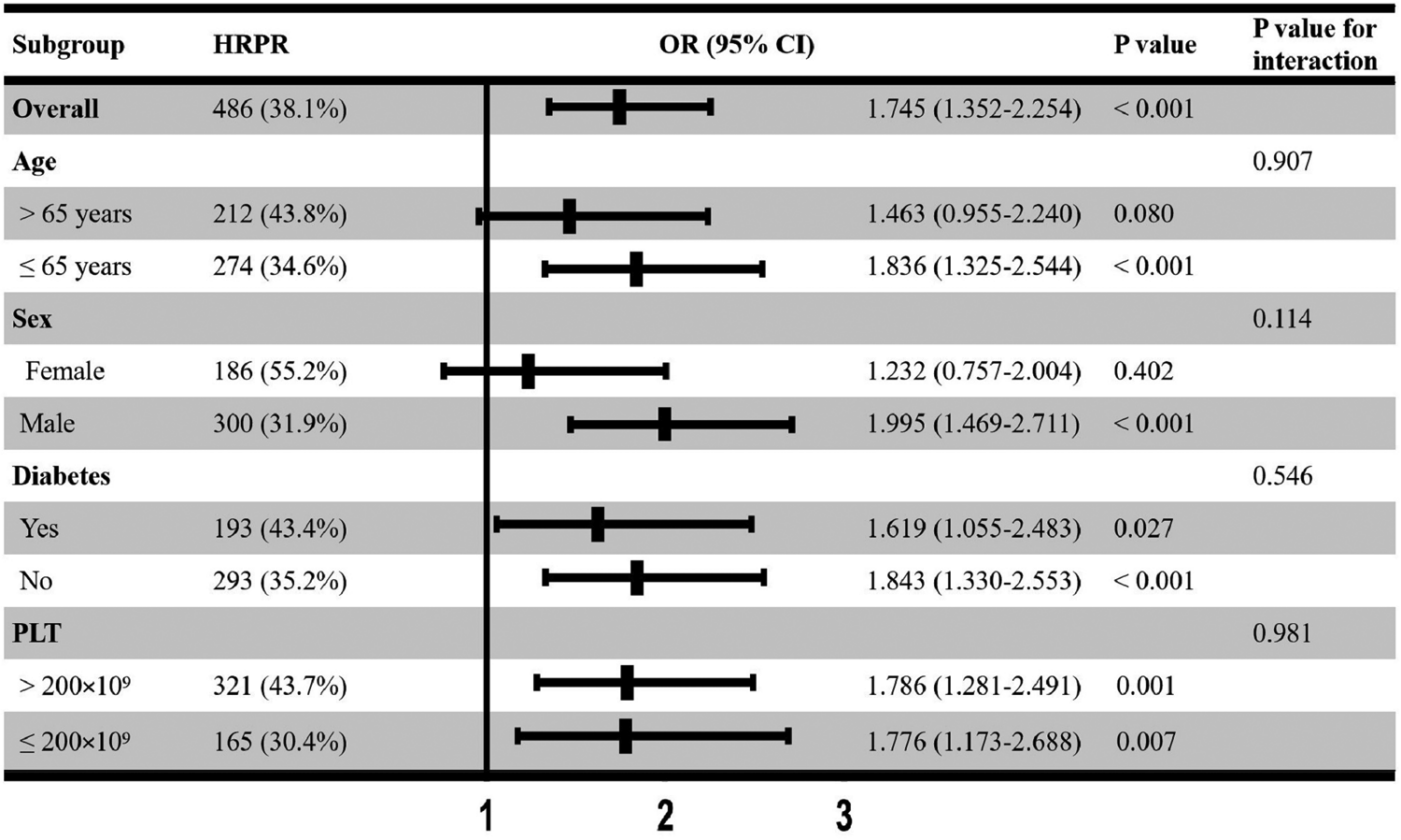
Subgroup analysis. OR, Odds ratio; CI, Confidence interval; HRPR, high residual platelet reactivity; PLT, platelet count.

Aiming to test the stability of results, we additionally performed subgroup analyses based on age (≤65 years or >65 years), sex, diabetes, and PLT count (≤200 × 10^9^ or >200 × 10^9^). As shown in Figure 3, the association of FFAs with HRPR showed no significant interaction with sex, age, diabetes, and PLT count (all *P*-values for interaction >0.05). However, it was worth noting that higher FFAs lost its significant association with HRPR in the female (*P* = 0.402) and >65 years (*P* = 0.080) subgroup and the interaction between FFAs level and sex required attention (*P* = 0.114). After PSM, there were 415 matched patients in both groups. Based on the new population, we further test the stability of the results using univariable and multivariable logistic regression models (Supplementary Material S2), and higher FFAs remained a significant independent factor associated with elevated HRPR risk (Model 3: OR = 1.765, 95% CI, 1.319–2.360). Based on 768 patients who had tested CYPC19*2, CYPC19*3, and CYPC19*17 alleles, we again conducted univariable and multivariable logistic regression models. We found higher FFAs was still associated with the occurrence of HRPR after adjusting for potential confounders including metabolizer status (crude OR, 95%CI, 1.534 (1.136–2.073); adjusted OR, 95%CI, 1.522 (1.074–2.158), showed in Supplementary Material S1, Table S2).

## 4. Discussion

The current study was a cross-sectional study based on a prospectively-collected database. We detected the effect of higher FFAs on ADP-induced residual platelet activity among patients using clopidogrel, and we demonstrated higher FFAs as an independent factor associated with elevated HRPR risk.

Clopidogrel is an essential component of secondary prevention medication prescription among patients with CAD (5). There are many proven risk factors associated with CAD, including insulin resistance, diabetes mellitus, dyslipidemia, chronic inflammation, and so on. Previous studies have suggested that some risk factors for CAD affect the antiplatelet efficacy of clopidogrel (22–24). FFAs is a vital energy source for most body tissues, which is predominantly produced from lipolysis from stored triglycerides. FFAs is metabolized through β-oxidation and converted to water and CO2 to produce large amounts of energy in the form of ATP via β-oxidation and the citric acid cycle in the mitochondria (25). So, the concentration of FFAs is influenced by rates of lipolysis and consumption, which can more sensitively reflect abnormal fat metabolism. The risk for metabolic disease development is worsened by high plasma FFAs content. Previous studies had found that elevated FFAs is a risk factor for many states of abnormal metabolism, including body obesity, insulin resistance, diabetes, hypertension, and non-alcoholic fatty liver disease(26). Our results also showed associations of higher FFAs with higher BMI, diabetes, hypertension, and abnormal lipid indicators. Previous studies have found that elevated FFAs may be closely associated with the occurrence, severity, and adverse prognosis of CAD (15–17). Morbid pathological states or diseases mentioned above were wildly acknowledged as CAD's risk factors and comorbidity, and the impact on these of FFAs might be part of the mechanism that abnormal metabolism cause CAD. To the best of our knowledge, whether higher FFAs affects the antiplatelet efficacy of clopidogrel remained unknown. Our findings suggested a novel potential clinical application of FFAs in predicting clopidogrel efficacy.

TEG dynamically measures physical parameters of clot formation, clot strength, and clot degradation to obtain a quantitative analysis of platelet function (19). MA_ADP_ reflects the strength of platelet-fibrin clots formed via the ADP pathway, which had a good diagnostic value for HRPR and a good prognostic utility for thrombosis and ischemic events among patients undergoing PCI (27). The current consensus defines MA_ADP_ > 47 mm as HRPR (20). ADP_i_ is calculated according to the equation $\mathrm{AD}P_{i}=\frac{MA_{\mathrm{ADP}}-MA_{\mathrm{fibrin}}}{MA_{\mathrm{thrombin}}-MA_{\mathrm{Fibrin}}}$, which reflects the extent of platelet inhibition. According to current consensus and previous Chinese study (11, 21), our study set HRPR as MA_ADP_ > 47 mm plus ADP_i_ < 50%. After the univariate and multivariate logistic regression analyses, higher FFAs was demonstrated as a potent independent factor associated with HRPR. Subgroup and sensitivity analyses further demonstrated that result was largely robust except for the female subgroup (*P* = 0.402). After adjusting confounders including clopidogrel's metabolizer status, we found that higher FFAs was still associated with HRPR. As previously reported, intermediate metabolizers and poor metabolizers of clopidogrel were more likely to present HRPR.

With stronger antiplatelet efficacy, novel P2Y12 antagonists, prasugrel and ticagrelor, have been widely used among patients with acute coronary syndrome or high thrombotic risk (4, 5). But, in many clinical scenarios, clopidogrel is still indispensable by virtue of the advantages of lower economic burden, lower bleeding risk, and fewer side effects, especially among patients with SCAD (4, 5). As a prodrug, clopidogrel can't exert the antiplatelet efficacy until being metabolized by cytochrome P450 (CYP450). Many factors have been reported to affect clopidogrel's antiplatelet efficacy, including genetic factors, drug-drug interaction, and biological and clinical factors. Of concern is the higher prevalence of the CYP2C19 loss-of-function alleles in east Asian populations (28), which leads to a higher incidence of HRPR. Evaluating antiplatelet efficacy using genetic testing or platelet function test is a common practice, although they are not recommended according to the guidelines. Accompanied by a higher proportion of HRPR, East Asians have been suggested to have a higher bleeding risk during receiving novel P2Y12 antagonists (29). TALOS-AMI trial (30) provided evidence that immediate switching from potent P2Y12 inhibitor to clopidogrel after the acute phase is non-inferior for patients suffering from acute myocardial infarction and significantly reduces bleeding risk compared to continued novel P2Y12 antagonists. The de-escalation strategy of DAPT is recommended to be guided based on the genotyping or platelet function test (20). Measuring blood biomarkers such as FFAs is more time-saving, easy to perform, and cheap than sequencing variants of cytochrome P450 genes. From a more practical clinical viewpoint, we provided an economical and simple indicator to assist in predicting HRPR and guiding DAPT de-escalation.

The explanations for the above results are not clear but may be multifactorial. As reported by previous studies, the current study also detected some factors associated with HRPR, including sex, age, diabetes, elevated hs-CRP, and so on. Some of these are associated with higher FFAs, which might partly explain the correlation between FFAs and HRPR. After adjusting for potential confounders and mediator variables, higher FFAs remained independently associated with HRPR, which meant FFAs itself affects residual platelet activity ignited via the ADP pathway. In an earlier study, Hoak JC et al. (31) found a high concentration of FFAs might enhance *in vitro* platelets' responsiveness to ADP. Patients with metabolic syndrome might enter into a hypercoagulable state. FFAs, a biomarker associated with metabolic syndrome, had been considered to be related to the hypercoagulable prothrombotic tendency in some basic research. Plasminogen activator inhibitor 1 is a biomarker of thrombosis, which exerts anti-fibrinolytic properties by inhibiting tissue-type plasminogen activators and urokinase-like plasminogen activators (32). Mathew M et al. found (33) that elevated FFAs within the physiological range induces plasminogen activator inhibitor 1, which maybe implies elevated FFAs as a pathogenic mechanism for thrombogenesis. TM-endothelial protein C receptor (EPCR)—Protein C pathway is a physiological anticoagulation system (34), Xie W et al. (35) found FFAs inhibit TM-EPCR-Protein C system via activating JNK signaling to promote the hypercoagulable state. Previous studies got evidence that FFAs promotes ADP-induced platelet aggregation and enhances the hypercoagulable state, which provides possible explanations for current observations. Among the study population, higher FFAs significantly enhances ADP-induced platelet activity quantified by TEG parameters and was demonstrated as an independent factor associated with HRPR while using clopidogrel. Current results call for more attention to FFAs while formulating antiplatelet prescriptions and evaluating antiplatelet efficacy of clopidogrel for SCAD patients undergoing PCI. In this research, we firstly found the association of FFAs with ADP-induced residual platelet reactivity among CAD patients receiving clopidogrel therapy. Without adding additional assays and medical expenditures, such as testing genes associated with clopidogrel resistance and testing platelet function, exploring biomarkers associated HRPR from routine items is clinically important. As a cheap and convenient indicator, FFAs might help to identify HRPR patients and to guide the formulation of antiplatelet prescriptions via improving existing prediction models or developing new models companied with other biomarkers.

However, the present study had several limitations. The current research was an observational study based on a single-center and small sample population, which might limit the generalization of our results and impose potential bias. And the cause-and-effect association cannot be determined. Second, due to the limited condition, we had not examined genotypes associated with the metabolism and transport of clopidogrel, such as CYP2C19 and ABCB1 genotypes. Third, our cross-sectional study lacked long-term follow-up data about ischemic and thrombosis events. Fourth, TEG parameters were derived from *in vitro* simulated coagulation process and might not accurately evaluate *in vivo* platelet activity. Fifth, all laboratory indicators were tested at baseline only once. Additionally, FFAs contains a range of different components, and different components of FFAs have different metabolic pathways and might be affected to various degrees by abnormal metabolism and other factors. The enzymatic colorimetric method we took just measured the total concentration of FFAs but not the concentration and proportion of FFAs constituents, which might lead to the loss of some crucial information. Finally, the biological mechanism of current conclusion is still unclear, and further investigation is warranted to explore the detailed mechanism.

## 5. Conclusion

Our research found that higher FFAs enhances ADP-induced residual platelet reactivity among CAD patients using clopidogrel and higher FFAs is independently associated with clopidogrel HRPR. The current finding suggested that FFAs is a new, reliable, and accessible clinical biomarker applied in assisting in predicting clopidogrel HRPR and helping to guide the choice of P2Y12 receptor antagonist.

## Data Availability

The raw data supporting the conclusions of this article will be made available by the authors, without undue reservation.
