# Corrigendum: The association between higher FFAs and high residual platelet reactivity among CAD patients receiving clopidogrel therapy

**DOI:** 10.3389/fcvm.2023.1250733

**Published:** 2023-07-11

**Authors:** Zehao Zhao, Shutong Dong, Tienan Sun, Kangning Han, Xin Huang, Meishi Ma, Shiwei Yang, Yujie Zhou

**Affiliations:** ^1^ Department of Cardiology, Beijing Anzhen Hospital, Capital Medical University, Beijing, China; ^2^ Beijing Institute of Heart, Lung and Blood Vessel Disease, Beijing, China; ^3^ Beijing Key Laboratory of Precision Medicine of Coronary Atherosclerotic Disease, Clinical Center for Coronary Heart Disease, Capital Medical University, Beijing, China

**Keywords:** free fatty acids (FFA), coronary artery disease, clopidogrel, platelet reactivity, thromboelastogram, percutaneous coronary intervention

A Corrigendum on The association between higher FFAs and high residual platelet reactivity among CAD patients receiving clopidogrel therapy By Zhao Z, Dong S, Sun T, Han K, Huang X, Ma M, Yang S and Zhou Y. (2023) Front. Cardiovasc. Med. 10:1115142. doi: 10.3389/fcvm.2023.1115142

**Incorrect Funding**

In the published article, there was an error in the Funding statement. The correct Funding statement appears below.

**FUNDING**

This work was supported by grants from the National Key Research and Development Program of China (2017YFC0908800), Pilot Projects for Public Welfare Development of Beijing Municipal Medical Institute “Precise medicine and interventional diagnosis and treatment platform for coronary heart disease” (2019-3), Capital's Funds for Health Improvement and Research (CFH 2020-2-2063) and Beijing Municipal Natural Science Foundation (7202041).

The authors apologize for this error and state that this does not change the scientific conclusions of the article in any way. The original article has been updated.

