# Supplementary material for: The association between higher FFAs and high residual platelet reactivity among CAD patients receiving clopidogrel therapy
Source: Front Cardiovasc Med. 2023 May 26;10:1115142. doi: 10.3389/fcvm.2023.1115142 (PMC10250738; doi:10.3389/fcvm.2023.1115142)
Supplement: Supplementary file 1 [file Table1.docx]

Supplementary Material 1

**Table 1 and 2** in Supplementary Material 1 were created based on 768 patients who had tested CYPC19*2, CYPC19*3 and CYPC19*17 alleles

# Table 1. Baseline characteristics in patients with and without HRPR

| Variables | Total (n = 768) | non-HRPR (n = 478) | HRPR (n = 290) | p |
| --- | --- | --- | --- | --- |
| Age (years), Mean ± SD | 61.16 ± 9.42 | 60.07 ± 9.46 | 62.96 ± 9.08 | <0.001 |
| Female, n (%) | 232 (30.2%) | 100 (20.9%) | 132 (45.5%) | <0.001 |
| BMI (kg/m^2^), Mean ± SD | 25.18 (4.89) | 25.38 (4.41) | 24.84 (5.59) | 0.134 |
| SBP (mmHg), Mean ± SD | 128.52 ± 15.35 | 127.64 ± 14.96 | 129.98 ± 15.90 | 0.04 |
| DBP (mmHg), Mean ± SD | 74.80 ± 10.88 | 74.95 ± 10.92 | 74.55 ± 10.83 | 0.622 |
| Heart rate (bpm), Mean ± SD | 69.34 ± 7.56 | 69.31 ± 7.54 | 69.38 ± 7.61 | 0.907 |
| Current smoker, n (%) | 189 (24.6%) | 128 (26.8%) | 61 (21.0%) | 0.088 |
| Hypertension, n (%) | 468 (60.9%) | 280 (58.6%) | 188 (64.8%) | 0.100 |
| Diabetes, n (%) | 260 (33.9%) | 147 (30.8%) | 113 (39.0%) | 0.024 |
| Dyslipidemia, n (%) | 406 (52.9%) | 268 (56.1%) | 138 (47.6%) | 0.027 |
| Previous CABG, n (%) | 15 (2.0%) | 8 (1.7%) | 7 (2.4%) | 0.653 |
| Previous PCI, n (%) | 206 (26.8%) | 140 (29.3%) | 66 (22.8%) | 0.058 |
| Clopidogrel loading, n(%) | 526 (68.5%) | 316 (66.1%) | 210 (72.4%) | 0.081 |
| FFAs (mmol/L), Mean ± SD | 0.42 (0.21 | 0.40 (0.20 | 0.43 (0.22 | 0.039 |
| WBC (×10^9^/L), Mean ± SD | 6.72 ± 1.85 | 6.90 ± 1.91 | 6.43 ± 1.71 | 0.001 |
| PLT (×10^9^/L), Mean ± SD | 212.59 ± 53.29 | 205.03 ± 53.33 | 225.04 ± 50.91 | <0.001 |
| Hb (g/L), Mean ± SD | 140.34 ± 16.44 | 143.36 ± 16.35 | 135.35 ± 15.37 | <0.001 |
| ALT (mmol/L), Median [IQR] | 21.00 [15.00, 28.25] | 21.00 [16.00, 28.00] | 20.00 [14.25, 28.75] | 0.123 |
| AST (mmol/L), Median [IQR] | 21.00 [18.00, 25.00] | 21.00 [17.25, 25.00] | 20.00 [18.00, 25.00] | 0.772 |
| ALB (mmol/L), Mean ± SD | 42.20 ± 3.56 | 42.26 ± 3.37 | 42.10 ± 3.85 | 0.531 |
| eGFR (ml/min/1.73 m^2^), Median [IQR] | 95.00 [86.69, 100.68] | 95.57 [87.97, 101.47] | 93.22 [84.11, 98.73] | 0.001 |
| Creatine (μmol/L), Mean ± SD | 71.66 ± 19.01 | 72.72 ± 18.48 | 69.91 ± 19.76 | 0.046 |
| UA (μmol/L), Mean ± SD | 351.44 ± 90.36 | 354.25 ± 87.01 | 346.80 ± 95.60 | 0.268 |
| TG (mmol/L), Mean ± SD | 1.62 ± 1.00 | 1.64 ± 0.99 | 1.60 ± 1.01 | 0.588 |
| TC (mmol/L), Mean ± SD | 4.02 ± 0.96 | 3.96 ± 0.91 | 4.14 ± 1.03 | 0.011 |
| HDL-C (mmol/L), Mean ± SD | 1.09 ± 0.25 | 1.08 ± 0.25 | 1.12 ± 0.27 | 0.048 |
| LDL-C (mmol/L), Mean ± SD | 2.34 ± 0.79 | 2.31 ± 0.76 | 2.40 ± 0.82 | 0.12 |
| FBG (mmol/L), Median [IQR] | 5.64 [5.07, 6.84] | 5.63 [5.02, 6.91] | 5.64 [5.12, 6.71] | 0.356 |
| FIB (g/L), Mean ± SD | 3.14 ± 0.59 | 3.05 ± 0.56 | 3.30 ± 0.61 | <0.001 |
| hs-CRP (mmol/L), Median [IQR] | 1.15 [0.49, 3.00] | 1.08 [0.44, 2.69] | 1.26 [0.62, 3.65] | 0.011 |
| MA_ADP_ (mm), Median [IQR] | 43.25 [35.10, 50.95] | 37.00 [30.20, 41.77] | 54.05 [50.10, 58.40] | <0.001 |
| ADP_i_ (%), Mean ± SD | 37.02 ± 23.27 | 49.92 ± 19.02 | 15.75 ± 10.53 | <0.001 |
| CYP2C19*2, n (%) |  |  |  | 0.008 |
| AA | 68 (8.9%) | 37 (7.7%) | 31 (10.7%) |  |
| AG | 328 (42.7%) | 189 (39.5%) | 139 (47.9%) |  |
| GG | 372 (48.4%) | 252 (52.7%) | 120 (41.4%) |  |
| CYP2C19*3, n (%) |  |  |  | 0.007 |
| AA | 2 (0.3%) | 1 (0.2%) | 1 (0.3%) |  |
| GA | 89 (11.6%) | 42 (8.8%) | 47 (16.2%) |  |
| GG | 677 (88.2%) | 435 (91.0%) | 242 (83.4%) |  |
| CYP2C19*17, n (%) |  |  |  | 0.876 |
| CT | 19 (2.5%) | 11 (2.3%) | 8 (2.8%) |  |
| CC | 749 (97.5%) | 467 (97.7%) | 282 (97.2%) |  |
| Metabolizer status, n (%) |  |  |  | <0.001 |
| RM | 12 (1.6) | 9 (1.9) | 3 (1.0) |  |
| NM | 295 (38.4) | 207 (43.3) | 88 (30.3) |  |
| IM | 365 (47.5) | 217 (45.4) | 148 (51.0) |  |
| PM | 96 (12.5) | 45 (9.4) | 51 (17.6) |  |
| CYP2C19 genotypes |  |  |  | <0.001 |
| 1*/17* (RM) | 12 (1.6%) | 9 (1.9%) | 3 (1.0%) |  |
| 1*/1* (NM) | 295 (38.4%) | 207 (43.3%) | 88 (30.3%) |  |
| 1*/2* (IM) | 296 (38.5%) | 180 (37.7%) | 116 (40.0%) |  |
| 1*/3* (IM) | 62 (8.1%) | 35 (7.3%) | 27 (9.3%) |  |
| 2*/17* (IM) | 6 (0.8%) | 2 (0.4%) | 4 (1.4%) |  |
| 3*/17* (IM) | 1 (0.1%) | 0 | 1 (0.3%) |  |
| 2*/2* (PM) | 68 (8.9%) | 37 (7.7%) | 31 (10.7%) |  |
| 2*/3* (PM) | 26 (3.4%) | 7 (1.5%) | 19 (6.6%) |  |
| 3*/3* (PM) | 2 (0.3%) | 1 (0.2%) | 1 (0.3%) |  |

HRPR, high residual platelet reactivity; BMI, body mass index; SBP, systolic blood pressure; DBP, diastolic blood pressure; PCI, percutaneous coronary intervention; CABG, coronary artery bypass graft; FFAs, free fatty acids; WBC, white blood cell count; PLT, platelet count; Hb, hemoglobin; ALT, alanine aminotransferase; AST, aspartate aminotransferase; ALB, albumin; eGFR, estimated glomerular filtration rate; UA, uric acid; TC, total cholesterol; TG, triglyceride; LDL-C, low density lipoprotein cholesterol; HDL-C, high density lipoprotein cholesterol; FBG, fasting blood glucose; FIB, fibrinogen; hs-CRP, high-sensitivity C-reactive protein; MAADP, Maximum amplitude of ADP-induced clot strength; ADPi, ADP-induced platelet inhibition rate; IM, intermediate metabolizers; NM, normal metabolizers; PM, poor metabolizers; RM, rapid metabolizers.

# Table 2: Univariable and multivariable logistic regression models

| Variables |  | OR (95% CI) | | | |
| --- | --- | --- | --- | --- | --- |
|  | **Crude Model** | **Model 1** | **Model 2** | **Model 3** | **Model 4** |
| FFAs > 0.445mmol/L | 1.534 (1.136-2.073) | 1.528 (1.126-2.072) | 1.532 (1.115-2.105) | 1.513 (1.093-2.095) | 1.522 (1.074-2.158) |
| Metabolizer status (compared to normal metabolizer) |  |  |  |  |  |
| Rapid metabolizer |  | 0.756 (0.199-2.878) | 0.534 (0.132-2.153) | 0.531 (0.130-2.165) | 0.914 (0.220-3.796) |
| Intermediate metabolizer |  | 1.573 (1.134-2.182) | 1.685 (1.197-2.374) | 1.650 (1.167-2.332) | 1.850 (1.279-2.676) |
| Poor metabolizer |  | 2.674 (1.664-4.298) | 2.858 (1.742-4.690) | 2.820 (1.704-4.667) | 2.963 (1.739-5.047) |
| Age | - |  | 1.027 (1.009-1.044) | 1.031 (1.013-1.050) | 1.034 (1.013-1.056) |
| Female | - |  | 2.932 (2.099-4.096) | 2.957 (2.072-4.220) | 1.848 (1.141-2.994) |
| Diabetes | - |  | - | 1.179 (0.842-1.651) | 1.232 (0.857-1.770) |
| Hypertension | - |  | - | 1.018 (0.733-1.413) | 1.019 (0.716-1.450) |
| Current smoker | - |  | - | 1.144 (0.770-1.700) | 1.179 (0.768-1.809) |
| Previous PCI | - |  | - | 0.640 (0.442-0.928) | 0.733 (0.493-1.090) |
| Clopidogrel loading |  |  |  | 1.132 (0.797-1.606) | 1.122 (0.774-1.626) |
| Hb | - |  | - | - | 0.981 (0.968-0.993) |
| WBC | - |  | - | - | 0.780 (0.698-0.871) |
| PLT | - |  | - | - | 1.007 (1.004-1.011) |
| LDL-C | - |  | - | - | 1.052 (0.841-1.317) |
| HDL-C | - |  | - | - | 0.870 (0.430-1.760) |
| ALT | - |  | - | - | 1.017 (1.005-1.029) |
| Creatine | - |  | - | - | 1.000 (0.990-1.010) |
| FIB | - |  | - | - | 2.483 (1.705-3.616) |
| hs-CRP | - |  | - | - | 0.952 (0.910-0.996) |

Model 1: adjusted for metabolizer status, Model 2: adjusted for sex, age, and variables included in Model 1, Model 3: adjusted for the medical history of hypertension, diabetes, and previous PCI, current smoker, usage pattern of clopidogrel, and variables included in Model 2, and Model 4: adjusted for Hb, WBC, PLT, HDL-C, LDL-C, ALT, creatine, FIB, hs-CRP, and variables included in Model 3.
OR, Odds ratio; CI, Confidence interval; HRPR, high residual platelet reactivity; FFAs, free fatty acids; PCI, percutaneous coronary intervention; Hb, hemoglobin; WBC, white blood cell count; PLT, platelet count; LDL-C, low density lipoprotein cholesterol; HDL-C, high density lipoprotein cholesterol; ALT, alanine aminotransferase; FIB, fibrinogen; hs-CRP, high-sensitivity C-reactive protein.
