# Supplementary material for: The association between higher FFAs and high residual platelet reactivity among CAD patients receiving clopidogrel therapy
Source: Front Cardiovasc Med. 2023 May 26;10:1115142. doi: 10.3389/fcvm.2023.1115142 (PMC10250738; doi:10.3389/fcvm.2023.1115142)
Supplement: Supplementary file 2 [file Table2.docx]

Supplementary Material 2

# Univariable and multivariable logistic regression models based on matched data

| Variables | OR (95% CI) | | | |
| --- | --- | --- | --- | --- |
|  | **univariate logistic regression** | **multivariate logistic regression** | | |
|  | **Crude Model** | **Model 1** | **Model 2** | **Model 3** |
| FFAs > 0.445mmol/L | 1.744 (1.321-2.304) | 1.736 (1.314-2.295) | 1.741 (1.314-2.308) | 1.765 (1.319-2.360) |
| Age | 1.012 (0.998-1.027) | 1.011 (0.996-1.026) | 1.011 (0.995-1.026) | 1.012 (0.995-1.028) |
| Female | 1.121 (0.833-1.509) | 1.057 (0.780-1.432) | 1.039 (0.752-1.436) | 1.081 (0.719-1.627) |
| Diabetes | 0.921 (0.696-1.220) | - | 0.875 (0.657-1.166) | 0.868 (0.648-1.161) |
| Hypertension | 1.156 (0.872-1.532) | - | 1.069 (0.798-1.431) | 1.074 (0.795-1.449) |
| Current smoker | 0.860 (0.631-1.174) | - | 0.955 (0.677-1.346) | 0.944 (0.667-1.337) |
| Previous PCI | 0.964 (0.709-1.310) | - | 0.947(0.689-1.301) | 0.934 (0.674-1.292) |
| Clopidogrel loading | 0.990 (0.754-1.301) |  | 1.019 (0.768-1.351) | 1.023 (0.768-1.362) |
| Hb | 1.001 (0.992-1.010) | - |  | 1.002 (0.991-1.013) |
| WBC | 0.995 (0.920-1.075) | - |  | 1.011 (0.925-1.104) |
| PLT | 1.000 (0.997-1.002) | - |  | 1.000 (0.997-1.003) |
| LDL-C | 0.965 (0.817-1.141) | - |  | 0.945 (0.789-1.132) |
| HDL-C | 1.043 (0.614-1.772) | - |  | 0.838 (0.468-1.502) |
| ALT | 1.000 (0.990-1.009) | - |  | 1.001 (0.991-1.011) |
| Creatine | 0.999 (0.991-1.006) | - |  | 0.999 (0.991-1.008) |
| FIB | 0.947 (0.749-1.198) | - |  | 0.911 (0.670-1.239) |
| hs-CRP | 0.998 (0.972-1.026) | - |  | 1.003 (0.969-1.039) |

After PSM, there were 415 matched patients in both groups. Based on the new population, we further test the stability of the results using univariable and multivariable logistic regression models. The table shows OR and 95% CI of higher FFAs based on the matched data.

Model 1: adjusted for sex and age, Model 2: adjusted for the medical history of hypertension, diabetes, and previous PCI, current smoker, usage pattern of clopidogrel, and variables included in Model 1, and Model 3: adjusted for Hb, WBC, PLT, HDL-C, LDL-C, ALT, creatine, FIB, hs-CRP, and variables included in Model 2.
OR, Odds ratio; CI, Confidence interval; HRPR, high residual platelet reactivity; FFAs, free fatty acids; PCI, percutaneous coronary intervention; Hb, hemoglobin; WBC, white blood cell count; PLT, platelet count; LDL-C, low density lipoprotein cholesterol; HDL-C, high density lipoprotein cholesterol; ALT, alanine aminotransferase; FIB, fibrinogen; hs-CRP, high-sensitivity C-reactive protein.
