# Supplementary figures and images for: The association between higher FFAs and high residual platelet reactivity among CAD patients receiving clopidogrel therapy
Source: Front Cardiovasc Med. 2023 May 26;10:1115142. doi: 10.3389/fcvm.2023.1115142 (PMC10250738; doi:10.3389/fcvm.2023.1115142)

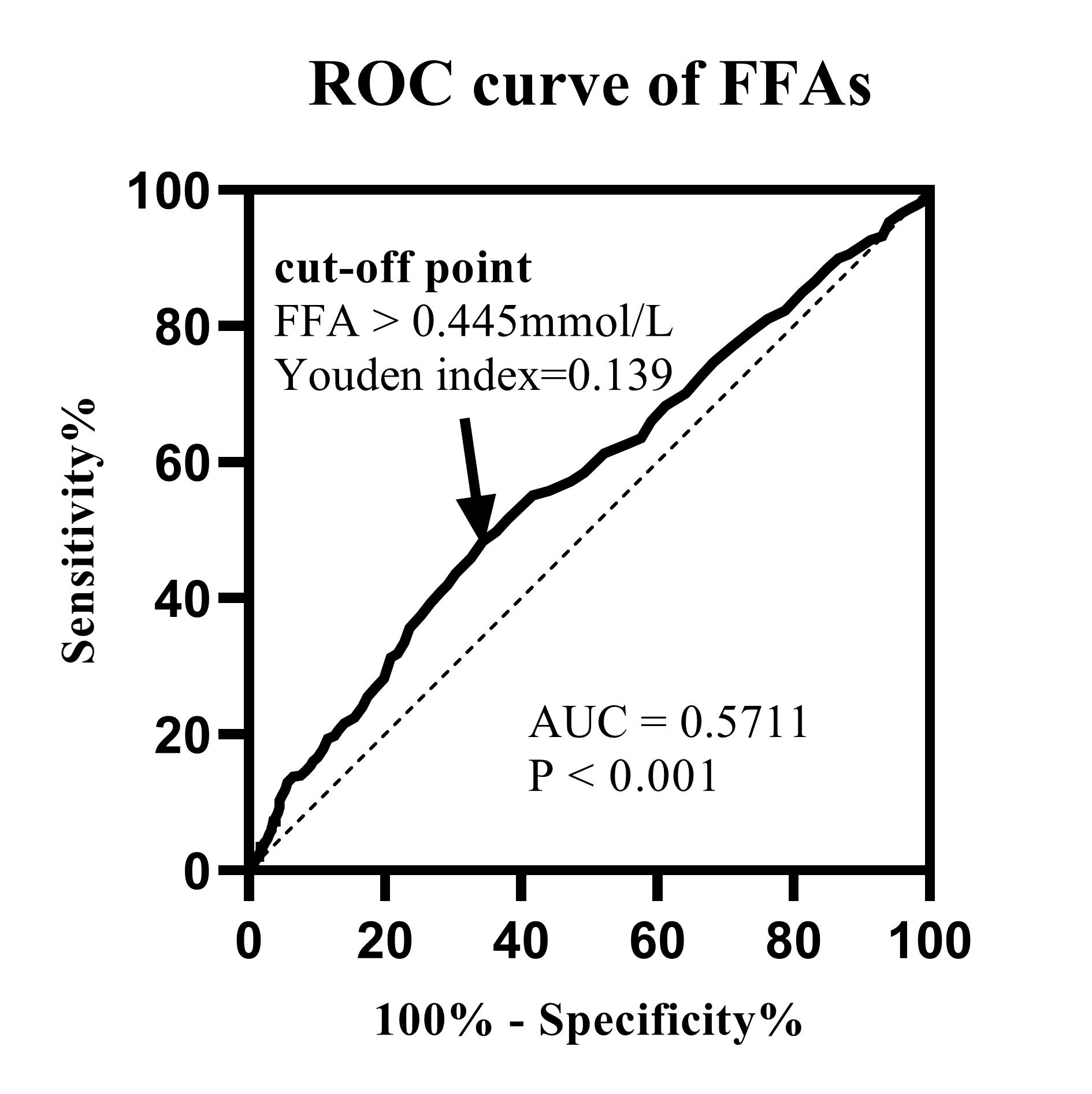

Supplement: Supplementary file 3 [file Image1.jpg]
